# Challenges in the Management of a Calvarial Defect in an NF1-Patient

**DOI:** 10.3390/diseases12120325

**Published:** 2024-12-12

**Authors:** Imane Abbas, Jinan Behnan, Abhishek Dubey, Genesis Liriano, Oren Tepper, Andrew J. Kobets

**Affiliations:** 1 The Leo M. Davidoff Department of Neurological Surgery, Albert Einstein College of Medicine, Montefiore Medical Center, Bronx, NY 10467, USA; jinan.behnan@einsteinmed.edu (J.B.); akobets@montefiore.org (A.J.K.); 2 Department of Microbiology and Immunology, Albert Einstein College of Medicine, Bronx, NY 10461, USA; 3 Department of Plastic Surgery, Albert Einstein College of Medicine, Montefiore Medical Center, Bronx, NY 10467, USA

**Keywords:** neurofibromatosis type 1, calvarial defect, cranioplasty

## Abstract

Background: Calvarial defects in NF1 are rare and lack standardized management guidelines. This study seeks to shed light on calvarial defects in NF1 patients with extensive skull erosion. Methods: This case report focuses on clinical and radiological presentations and surgical interventions during six years of follow-up, comparing the results with those in the literature. Results: A five-year-old female with NF1 disease was diagnosed with a spontaneous calvarial defect in the occipital region and an arachnoid cyst underneath. The lesion enlarged progressively over the years and at the age of nine, she underwent her first surgery. Our surgery team performed a cranioplasty using a split-thickness bone graft harvested from the parietal bone after cyst decompression. Two years later, she underwent revision surgery with a titanium mesh plate due to total resorption of the initial bone graft and unsuccessful closure of the large defect. Conclusions: Calvaria defects are a relatively unknown aspect of NF1, and no standard treatment exists. Their management requires a personalized approach, considering factors like lesion size, and the potential for multiple interventions throughout the patient’s lifetime. Due to their progressive nature and the possibility of additional lesions, long-term follow-up is crucial for effective monitoring and intervention planning.

## 1. Introduction

Calvarial defects are very rare manifestations in patients with neurofibromatosis type 1. NF1 is one of the most common genetic disorders and tumor predisposition syndromes inherited by an autosomal dominant mode. It affects both the nervous system and the skin, often displaying multi-systemic abnormalities [1]. The prominent bone anomalies typically associated with NF1 include scoliosis, sphenoid wing dysplasia, and tibial pseudoarthrosis, which are hallmarks of diagnosis [1,2]. Although several skull defects have been described, out of those, calvarial defects are a rare occurrence. The most reported defects are localized within the lambdoid suture region, and the majority of them are associated with overlying plexiform neurofibromas and/or dural ectasia.

Many techniques of cranioplasty utilizing either a titanium material or a bone graft have been used for reconstructive purposes [3,4,5,6,7]. The cranioplasty approach is highly personalized and takes into account factors such as the patient’s age, the specific characteristics of the defect, the progressive nature of the bone lesion, and other clinical considerations. This report outlines these considerations and options for calvarial defect management in an exemplary case managed at our institution.

## 2. Materials and Methods

A standardized case report form was used to extract data from our institutional database for our patient who was treated first in 2018 and underwent a second surgery in 2020.

## 3. Results

**Figure 1:**
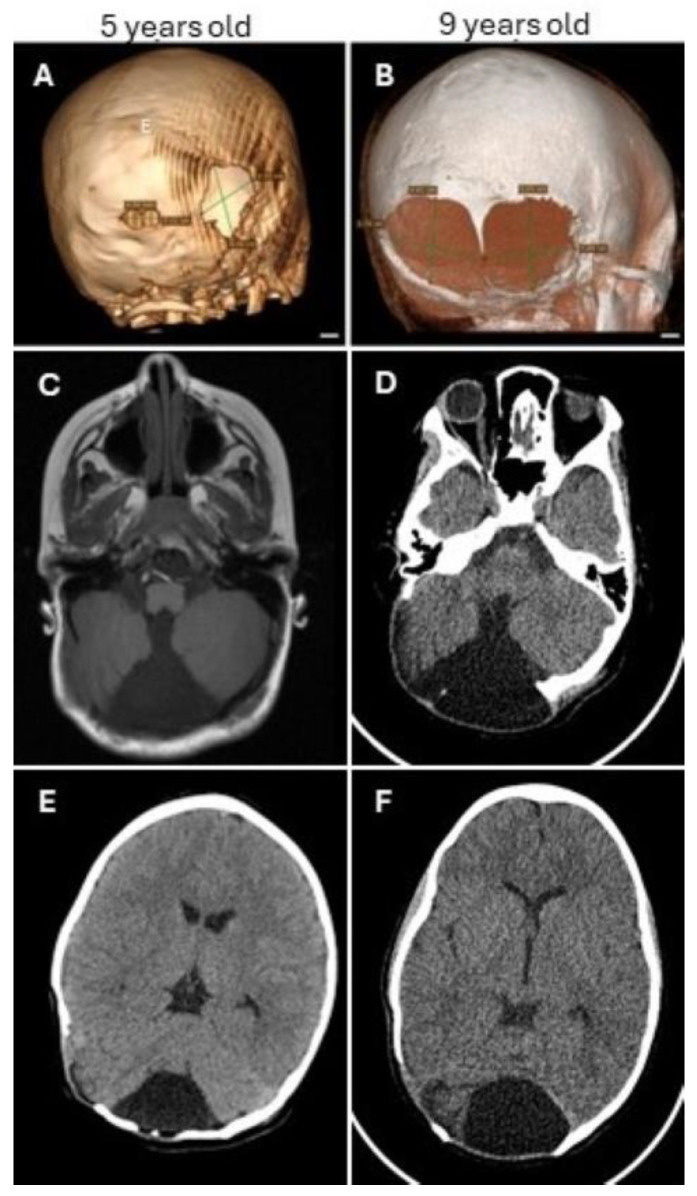
(**A**,**B**) Three-dimensional CT images demonstrating the progression of the bone defect for four years in the right occipital skull extending along and posterior to the lambdoid suture, at 5 years old (**A**) and 9 years old (**B**). (**C**,**E**) Images obtained at 5 years old: (**C**) axial T1 MRI demonstrating a large posterior fossa cyst displacing the cerebrum, and (**E**) axial CT image showing the bone defect in the right occipital skull along the lambdoid suture with an underlying mild bulge of the dura and brain parenchyma through the defects. A smaller defect is present in the posterior central part of the occipital bone. (**D**,**F**) Axial CT images obtained at 9 years old show the enlargement of both the defect and the cyst.

The case involves a 5-year-old female with a family history of neurofibromatosis type 1 (NF1) inherited from her mother. She was referred by a pediatrician for an evaluation of a posterior fossa cyst and a posterior fossa defect. Clinical findings revealed a palpable soft area on the occipital region of her head. She experiences no symptoms, including numbness, weakness, imbalance, vomiting, or other focal neurological changes. The brain imaging demonstrated two calvarial defects in the occipital bone, one extending along and posterior to the lambdoid suture, and underneath, an arachnoid cyst within the cisterna magna, which communicated with the fourth ventricle and harbored a large amount of cerebrospinal fluid (Figure 1A,C,E). A reconstructive surgery for the defect was discussed, but the patient was lost to follow-up for 4 years.

Four years later, she consulted again and stated that tumefaction had been expanding and causing concern for cosmetic deformity. A new CT revealed a large extension of the calvarial defects in the occipital bone and to the parietal skull crossing the right lambdoid suture and a significant enlargement of the cyst (Figure 1B,D,F). Clinical examination showed a 7 cm wide, soft, palpable area in the occipital region with no pressure under. The patient underwent a cranioplasty using a split-thickness calvarial graft harvested from the overlying parietal bone. The surgery went well without complication, and the surgeon was able to only cover part of the defect, without clear contact with the bone edges. Absorbable plates (KLS Martin, Tuttlingen, Germany) were used to support the calvarial graft given the patient’s age and expectation for growth. The postoperative course was uncomplicated.

**Figure 2:**
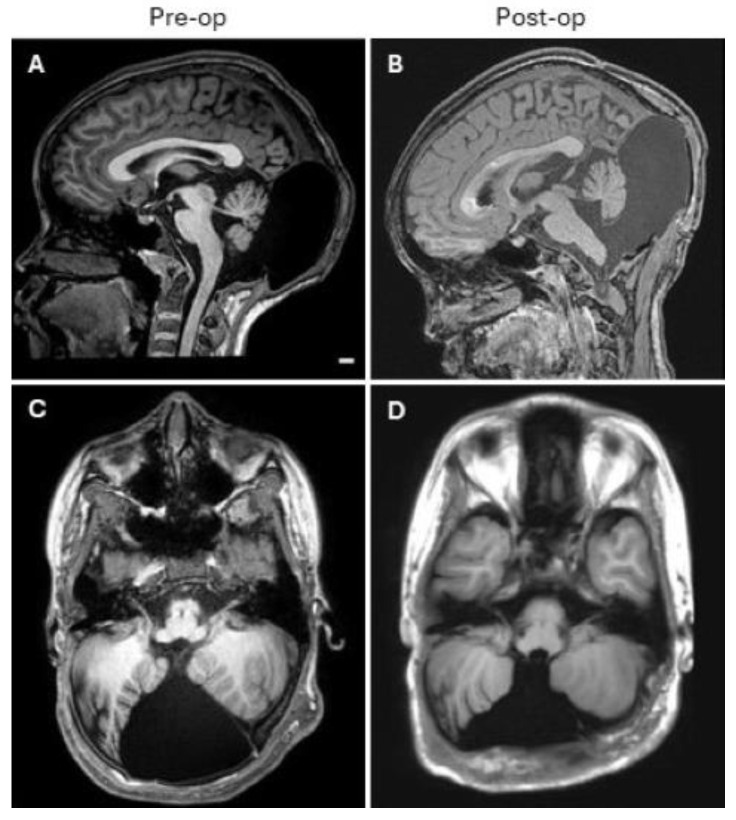
Sagittal and axial T1 MR imaging at 15 years of age. (**A**,**C**) Before the second surgery, it shows a large arachnoid cyst of the cisterna magna protruding beyond the margins of the defect inferiorly. (**B**,**D**) One month later, it shows a decreased external herniation of the cyst and an increased soft tissue between the margin of the cyst cavity and the skin.

Two years after the preliminary surgery, she presented with regrowth of the cyst. Examination of the defect showed it to be readily palpable and compressible on the exam without clear pressure or tension, and her surgical scar was well healed. A new MRI was obtained (Figure 2A,C), which showed a continuous large midline defect and the absence of the calvarial graft implanted in the previous surgery. It appeared that all grafted material and absorbable plates had been resorbed. However, the donor sites for the graft parietally appeared intact. The patient underwent a second cranioplasty for the associated defect. Given the compressible expansion of the cystic fluid underneath there was a consideration for consequent elevation of intracranial pressure or hydrocephalus. Initial management focused on cyst decompression. This approach was also deemed essential to facilitate tension-free graft placement and to mitigate the likelihood of future cystic enlargement. Rather than moving to a shunt at this time, a neuroendoscopic cystic fenestration was elected. Her previous incision was opened along a 1 cm segment, and dissection proceeded down to the dura, which was opened, and a neuroendoscope was placed within. The region of the foramen magnum, supra cerebellar cistern, and ambient cistern were evaluated for fenestration; however, no safe option was deemed approachable. Therefore, the cyst was simply decompressed after the endoscope was removed by about 50%, and the dura was again closed and sealed. The remainder of the incision was opened to the level of the dura without additional breeches. The dura was slack at this point. Given the patient’s age and prior intervention, a titanium mesh cranioplasty was chosen to provide structural coverage and closure of the wound. Calvarial grafting already failed and was not a great option, and the team confirmed that a manufactured graft would be difficult to fit in this space. The graft was placed and was able to be secured using a titanium low-profile plating system superiorly to the parietal bone and laterally to the remaining occipital bone. The inferior edge of the defect was not opened, and the graft at its inferior point was about 1–2 cm from the inferior defect edge. Both large defects were covered for the majority of their size. The overlying tissues were closed in a watertight fashion, and cosmetic closure was achieved. An MRI was obtained postoperatively which demonstrated the stability of the cyst that was no longer herniating through the defect and the congruency of contour with the adjacent skull (Figure 2B,D).

The patient was followed serially without reported adverse events. At the two-year postoperative follow-up, there was no evidence of recurrent cyst expansion or graft protrusion. The surgical site maintained its initial postoperative anatomic configuration.

## 4. Discussion

Calvarial defects are notably rare in patients with NF1. Only a few cases, including ours, have been reported in the literature [3,4,5,6,7,8,9,10,11,12,13,14,15]. This rarity highlights the unique nature of each case.

### 4.1. Clinical Features and Diagnosis

The presentation of calvarial lesions in neurofibromatosis type 1 (NF1) patients is highly variable, influenced by lesion type and location. These lesions typically manifest in childhood, with peak incidence reported in the pediatric population [3,4,5,6,7,8,9,10,11,12,13,14]. Clinical presentation and diagnosis can vary significantly. Small, isolated defects may increase the risk of misdiagnosis or be incidentally discovered during routine NF1 imaging studies. Large defects may exhibit palpable bone thinning and compliance [5,8,10]. However, most lesions are intimately associated with overlying or underlying structures; specific lesion types include plexiform neurofibromas [3,4,6,10,11], an atypical meningioma [12], a dural ectasia, and a cyst or brain herniation [8,9,10,11] potentially complicating diagnosis.

Three-dimensional computed tomography (CT) is indispensable for the precise delineation and characterization of calvarial defects. Magnetic resonance imaging (MRI) is essential for the comprehensive evaluation of associated soft tissue abnormalities and preoperative planning.

### 4.2. Bone Defect Characteristics

Calvarial defects exhibit a predilection for specific regions of the skull vault. A preponderance of osseous defects is observed in the vicinity of the lambdoid suture [4,5,8,11,13,14]. The present case features two substantial bony lesions, one of which is distinctly localized to the right lambdoid suture. The pathophysiology underlying calvarial defects and their frequent association with the lambdoid suture remains enigmatic.

These calvarial defects are characterized by their progressive nature, with lesions growing over time and the potential appearance of additional defects [10,11]. In our patient, the bone graft was totally resorbed, and the defect was enlarged two years later. Furthermore, the bony margins appeared irregular and without lateral predominance. In fact, during bone healing, the natural evolution of the bone graft materials is the internalization of the graft and bone regeneration after a few months; however, the insufficiency in osteogenic activity and low osteoinductivity of bone grafting materials in NF1 reduce the ability for bone substitution, especially in large bone defects [16]. Previous research has linked NF1 loss to elevated Ras-GTP levels, stimulating osteoblast proliferation and impairing differentiation. Furthermore, increased Ras activity has been implicated in heightened osteolytic activity and subsequent bone resorption [17]. While the association with an arachnoid cyst, in this case, raises the possibility of the mechanical bone erosion of the skull due to direct contact with a cerebrospinal fluid (CSF)-filled cyst, this hypothesis may represent a contributing factor to bone resorption, rather than the sole etiology; the absence of hydrocephalus suggests that the cyst was not associated with elevated intracranial pressure, mitigating the likelihood of pressure-induced bone erosion as the primary mechanism.

The failure of bone integration leads to unsuccessful cranioplasties, and thereby difficulties managing these lesions, and in consequence, it underlines the need for consistent monitoring and interventions.

### 4.3. Reconstructive Surgery

The management of calvarial defects in neurofibromatosis type 1 (NF1) patients regarding the indication and timing of reconstructive cranioplasty is complex and necessitates careful consideration of multiple factors, particularly in the pediatric population. While a significant proportion of reported cases in the literature have been asymptomatic and did not necessitate surgical intervention [3,5,8,10,12,13,14,15], cranioplasty may be indicated in specific patient subsets; patients with palpable calvarial defects and increased risk factors for traumatic brain injury, such as those engaging in high-impact physical activity or with a history of head trauma, who may be prime candidates for surgical intervention. Additionally, the elevated incidence of epilepsy in neurofibromatosis type 1 (NF1) patients, which increases with age [18], can significantly influence treatment decisions. However, cranioplasty in the pediatric population presents unique challenges due to ongoing craniofacial growth and development. Implant placement in a dynamic environment can result in implant instability and potential skull remodeling.

In cases where calvarial defects are associated with overlying or underlying lesions, the surgical management becomes further complicated in several ways.

*The surgical Approach:* The presence of overlying lesions like plexiform neurofibromas may necessitate a more complex surgical approach to ensure complete removal or adequate management of these lesions while addressing the bone defect. This complexity increases the surgical risk and may require specialized expertise in neurosurgery or craniofacial surgery.

*Tissue Integrity:* Overlying lesions can compromise the integrity of adjacent tissues, including the scalp and subcutaneous tissues. This may necessitate extensive tissue resection and reconstruction. The manipulation or removal of these lesions during cranioplasty can lead to challenges in achieving proper tissue closure and wound healing post-surgery given that we need an adequate quality of soft tissue coverage of the bone grafting to prevent complications.

*The risk of Recurrence:* Some lesions associated with NF1, such as plexiform neurofibromas, arachnoid cysts, or brain tumors, tend to recur, as happened with our case. Surgical correction of the bone defect may be complicated by the need to perform further surgeries and the risk of reappearance or fault of integration of the bone graft previously performed.

A collaborative, multidisciplinary approach is essential for optimizing patient management and achieving optimal reconstructive outcomes [17].

### 4.4. Cranioplasty Materials

There is no standard algorithm, and the treatment is individualized for each case. Various techniques for cranioplasty have been reported in the literature.

*Autologous bone:* One patient in the literature received split-thickness iliac crest bone grafts and resorbable plating [11]. In our case, the patient had a split-thickness bone graft harvested from the parietal bone and secured with resorbable plates. In both cases, neither integration nor complete preservation of the bone grafts was observed, resulting in the resorption of the implanted material, leading us to think of the hypothesis of the primary hypoplasia in the skull; also, the presence of the cyst underneath may have aggravated the situation by exerting a mechanical force that contributes to bone erosion. Periosteum use represents another potential approach when the defect is small, although its efficacy and long-term outcomes remain uncertain due to the lack of follow-up data in the literature [6].

*Titanium and synthetic bone substitutes:* This includes the utilization of titanium mesh combined with methyl methacrylate [7,11], CAD/CAM prefabricated titanium plates, and optimized titanium implants [9]. While these plates offer improved stability by effectively closing the defect, their application presents challenges. A significant margin of the skull bone is required to prevent the thin, compromised regions usually present around the defect site. Furthermore, their use in pediatric patients necessitates caution due to the dynamic nature of cranial growth. The material properties of the plate could exert undue pressure, potentially leading to iatrogenic skull remodeling. This concern is particularly relevant given the predominance of patients under sixteen years of age within this treatment population [19].

**Follow-up Duration:** The need for multiple procedures in our case and some cases emphasize the importance of closely monitoring patients with calvarial defects and NF1. Adaptive interventions are crucial to addressing progressing issues and optimizing patient outcomes. Long-term follow-up is mandatory, as studies have reported recurrences even after a decade from the initial corrective procedure [11].

## 5. Conclusions

This study underscores the complexities and challenges that can arise in managing calvarial defects in patients with NF1. The decisions for the timing of intervention, the extent of intervention, and the materials used for treatment are all nuanced and complicated, requiring careful consideration to optimize outcomes for patients. The scarcity of these cases further underscores the need for additional research in this field to yield a better understanding of the molecular basis for these lesions, which may provide better treatment approaches for affected individuals in the future.

## Data Availability

No new data were created or analyzed in this study. Data sharing does not apply to this article.
